# Supplementary material for: Deletion of wfs1 Impairs Oligodendrocyte Precursor Cells Dorsal Distribution and Myelination Through the wfs1-hmgcs1 Axis in Zebrafish
Source: Biology (Basel). 2026 Aug 16;15(16):1402. doi: 10.3390/biology15161402 (PMC13510151; doi:10.3390/biology15161402)
Supplement: Supplementary file 1 [file biology-15-01402-s001.zip › File S1-Transcriptome sequencing data and differentially expressed genes (DEGs) in wfs1-- zebrafish at 5 dpf/1.GO/wfs1KOvsWT/GSEA/WT/PEROXISOMAL_MEMBRANE/PEROXISOMAL_MEMBRANE(GO_0005778).html]

Details for gene set PEROXISOMAL\_MEMBRANE(GO:0005778)[GSEA]


|  || Dataset | deg.sample |
| Phenotype | sample.cls |
| Upregulated in class | WT |
| GeneSet | PEROXISOMAL\_MEMBRANE(GO:0005778) |
| Enrichment Score (ES) | -0.47380874 |
| Normalized Enrichment Score (NES) | -1.6955029 |
| Nominal p-value | 0.0 |
| FDR q-value | 0.3152505 |
| FWER p-Value | 0.927 |
Table: GSEA Results Summary

  

Fig 1: Enrichment plot: PEROXISOMAL\_MEMBRANE(GO:0005778)
  
Profile of the Running ES Score & Positions of GeneSet Members on the Rank Ordered List

  

| PROBE | DESCRIPTION   (from dataset) | GENE SYMBOL | GENE\_TITLE | RANK IN GENE LIST | RANK METRIC SCORE | RUNNING ES | CORE ENRICHMENT || 1 | 566742 | pex11b |  |  | 3802 | 0.868 | -0.0722 | No |
| 2 | 563203 | pex11g |  |  | 3871 | 0.855 | -0.0278 | No |
| 3 | 100034578 | si:dkey-97m3.1 |  |  | 4169 | 0.799 | 0.0063 | No |
| 4 | 336430 | si:ch73-21k16.5 |  |  | 6579 | 0.530 | -0.0405 | No |
| 5 | 406829 | far1 |  |  | 7862 | 0.434 | -0.0572 | No |
| 6 | 566367 | abcd1 |  |  | 7979 | 0.425 | -0.0377 | No |
| 7 | 324778 | pex19 |  |  | 8696 | 0.388 | -0.0391 | No |
| 8 | 797988 | fis1 |  |  | 9600 | 0.327 | -0.0497 | No |
| 9 | 565760 | pex11a |  |  | 11543 | 0.214 | -0.0990 | No |
| 10 | 393197 | pex3 |  |  | 11738 | 0.204 | -0.0940 | No |
| 11 | 368672 | atad1a |  |  | 14412 | 0.073 | -0.1739 | No |
| 12 | 767748 | abcd4 |  |  | 14904 | 0.051 | -0.1865 | No |
| 13 | 368412 | atad1b |  |  | 15310 | 0.032 | -0.1975 | No |
| 14 | 793906 | pex1 |  |  | 16843 | -0.028 | -0.2441 | No |
| 15 | 393618 | pex13 |  |  | 17520 | -0.054 | -0.2624 | No |
| 16 | 573990 | pex16 |  |  | 18008 | -0.076 | -0.2735 | No |
| 17 | 541479 | hmgcrb |  |  | 18296 | -0.089 | -0.2777 | No |
| 18 | 393174 | pex12 |  |  | 18527 | -0.100 | -0.2795 | No |
| 19 | 559933 | pex14 |  |  | 19567 | -0.146 | -0.3041 | No |
| 20 | 792998 | pex6 |  |  | 22661 | -0.305 | -0.3846 | No |
| 21 | 100170781 | abcd3b |  |  | 22699 | -0.307 | -0.3691 | No |
| 22 | 572335 | gnpat |  |  | 23240 | -0.344 | -0.3673 | No |
| 23 | 555635 | fndc5b |  |  | 25304 | -0.467 | -0.4067 | No |
| 24 | 566065 | wu:fe02h09 |  |  | 26104 | -0.538 | -0.4025 | No |
| 25 | 449821 | pex10 |  |  | 26473 | -0.575 | -0.3828 | No |
| 26 | 334644 | pex26 |  |  | 29373 | -1.022 | -0.4183 | Yes |
| 27 | 559054 | hmgcra |  |  | 30946 | -1.905 | -0.3640 | Yes |
| 28 | 791920 | tmem35 |  |  | 31112 | -2.110 | -0.2545 | Yes |
| 29 | 100093708 | slc25a17 |  |  | 31340 | -2.507 | -0.1254 | Yes |
| 30 | 406803 | abcd3a |  |  | 31390 | -2.624 | 0.0157 | Yes |
Table: GSEA details
[plain text format]

  

Fig 2: PEROXISOMAL\_MEMBRANE(GO:0005778)
  
Blue-Pink O' Gram in the Space of the Analyzed GeneSet

  

Fig 3: PEROXISOMAL\_MEMBRANE(GO:0005778): Random ES distribution
  
Gene set null distribution of ES for
 **PEROXISOMAL\_MEMBRANE(GO:0005778)**
